# Exposure to chemical pollutants and biological aerosol in indoor facilities for recreational and sport horses

**DOI:** 10.1186/s12917-024-03930-2

**Published:** 2024-02-28

**Authors:** Izabela Rodzyń, Katarzyna Karpińska, Hanna Bis-Wencel, Łukasz Wlazło, Mateusz Ossowski, Katarzyna Strzelec, Sebastian Jaguszewski, Bożena Nowakowicz-Dębek

**Affiliations:** 1 Department of Animal Hygiene and Environmental Hazards, Faculty of Animal Sciences and Bioeconomy, University of Life Sciences in Lublin, Akademicka 13, Lublin, 20-950 Poland; 2 Department of Horse Breeding and Use, Faculty of Animal Sciences and Bioeconomy, University of Life Sciences in Lublin, Akademicka 13, 20‑950, Lublin, Poland; 3 Student Scientific Association of Occupational and Environmental Hazards, Faculty of Animal Sciences and Bioeconomy, University of Life Science in Lublin, Akademicka 13, 20‑950, Lublin, Poland

**Keywords:** Stables, Riding hall, Chemical hazards, Dust concentration, Bioaerosols

## Abstract

**Background:**

Due to the increasing prevalence of equine non-infectious respiratory disease, the air contamination in equine housing (Stables A-C) and training facilities (indoor riding arenas A - C) was investigated. The aim of the study was to monitor gaseous pollutants, bioaerosols, and dust concentrations at three different sites (stables and riding halls), where different floor materials were used in the riding halls.

**Materials and methods:**

Air quality was monitored in housing for horses and in riding halls in terms of dust concentration, the presence of gaseous chemical pollutants, and concentrations of biological aerosol. Statistical analysis was performed using analysis of variance (ANOVA). The levels obtained were compared with acceptable limits.

**Results:**

Among the gaseous pollutants identified, the highest concentration was obtained for ammonia in stables B and C (16.37 and 22.39 mg/m^3^, respectively). Standards for total dust were exceeded in stables B and C and in riding halls B and C. The highest numbers of bacteria and fungi were recorded in stables A and C and in riding hall B. *Ulocladium* sp. had the highest percentage share among the moulds identified.

**Conclusions:**

The results confirm that the wrong choice of bedding in the stable and indoor riding arenas may contribute, even in short training periods, to equine non-infectious respiratory disease (equine asthma). Bioaerosol suspended in the air together with released gaseous pollutants can exacerbate this phenomenon, which even in the case of short training periods can lead to equine asthma of varying degrees of severity. For this reason, the choice of floor material in riding halls should be treated as a priority, as the wrong decision can shorten the period during which the horse can be used for recreational purposes.

## Background

The crucial factors determining the health of horses include their housing system and the environmental conditions in indoor spaces (boxes, stables, and riding halls) [1, 2]. Staying in buildings with inadequate air exchange parameters and excessive concentrations of pollutants can lead to respiratory disease. It is particularly difficult to maintain optimal microclimate parameters in the autumn and winter, when the CO_2_ concentration and relative humidity in stables increase, which can increase the number of breaths taken by horses and impede adequate oxygenation of the body [3, 4]. Changes caused by bacterial enzymes in animal waste and feed residues lead to the release of ammonia, which irritates the lungs and impairs the immune system, causing immune incompetence in animals and increasing their susceptibility to microbiological pollution [5–7]. Dust concentration plays an important role in the spread of microbes in the air of indoor facilities for animals. In winter, the levels of total and respirable dust and (1.3)-β-D-glucan are higher than in summer [8]. These factors lead to an increase in neutrophil counts in the blood and in expression of IL-6 at the mRNA level. Researchers suggest monitoring markers of pulmonary inflammation in horses kept in traditional stables as markers of the state of the microclimate in indoor spaces [9].

The most common disease resulting from poor air quality is chronic aseptic inflammation called “equine asthma” [6, 10, 11]. Due to similar clinical symptoms to other diseases, it is necessary to diagnose respiratory system disorders that not only affect the health of horses but also their performance in various equestrian disciplines [11, 12]. According to Bond et al. [13], progressive respiratory inflammation in horses often stems from poor microclimatic conditions. Monitoring the air contamination in the stables is essential for the prevention of equine asthma and therefore is also substantial for their quality of life [14, 15]. To avoid the negative effects of microclimatic factors and poor-quality floor material (litter) on the health of horses and workers, indoor spaces that may have an aerogenic effect require monitoring. Therefore, a study was carried out to monitor released chemical pollutants and biological aerosol in stables and riding halls with varied microclimatic conditions and different types of floor material. The results may suggest to users about the microclimatic conditions and choice of floor material during recreational use of horses.

## Materials and methods

### Sampling sites

The study was carried out at three stables (SA, SB, and SC) and the indoor riding halls (RHA, RHB, and RHC). Each building varied in terms of its modernity and the timing of its modernization. The type of ventilation in all buildings was natural gravity ventilation.

Site A was the newest (built in 2005–2007), a wooden stable with boxes facing outward, housing 25 horses during the study period. Quartz sand was used as the floor material in their riding hall.

Site B – a decades-old stable (built in 1918) and riding hall (built in the 1970s), following slight modernization. The floor material in their riding hall was sand, regularly replenished and supplemented with sawdust. During the study there were 12 horses in the buildings.

Site C – a farm (stables were built in the 1960s, and the riding hall at the turn of the 20th century) where space was made for horses to stay in boxes, alongside other animals, and their indoor riding hall was filled with sand mixed with fine gravel. There were 10 horses in the stables during the study period.

The bedding material in all stables was straw. The study was carried out during a typical nominal workday, when these conditions prevail. ‘Standard’ may not be the best word. The samples were taken in close proximity to the horses, up to about 30 cm. The work day during which the study was conducted can be described as representative. The workers carried out their activities associated with handling of the animals or their use for recreational purposes.

### Chemical and biological analysis

Monitoring of microclimatic conditions was carried out in all examined facilities in the autumn and winter, when temperatures are lower and when breeders close the stable doors so it will not be too cold. Samples for analysis of gaseous pollutants in the air of three stables and riding halls were collected into Tedlar® bags (Sensidyne, Inc., Clearwater, USA) using an electric pump and then condensed on MX-06-2131 sorbent tubes (SKC Inc., Pennsylvania, USA). Then thermal desorption was carried out (Dynatherm, Analytical Instruments, Inc., Oxford, USA), and the sample was injected into a gas chromatograph with an FPD detector (HP 5890 series II, Hewlett Packard, Santa Clara, USA). Thermal desorption tubes were analysed by GC–MS (gas chromatography–mass spectrometry) using a thermal desorption system with a 0.25 μm FFAP column. Compounds were identified using mass spectra and retention times of reference standards and/or mass spectra from the NIST98 library (NIST, Gaithersburg, MD), at a match level of > 70%. Chromatograms of the gases were prepared in a permeation chamber heated to the optimal temperature for the tube. The peaks of the gases were identified on the basis of baselines, and the procedure and operation of the chromatographic system were identical for the reference and test samples. Compounds were quantified using external standards and estimates based on molecular weight and chemical class [16].

In the stables and riding halls, three half-hour measurements of the dust concentration were made by the gravimetric method using an SKC air sampler with an SKC IOM cassette (SKC Ltd, USA) and a GilAir 5 sampler (Sensidyne, Inc. Clearwater, USA) with an HD Cyclone head for respirable dust. The total and respirable dust fractions were measured using conditioned Whatman GF/A filters with a diameter of 25 mm (no. 1820-025) with air flow of 2 m/s^2^ and 2.2 m/s^2^, respectively. After each sampling the cassette was replaced. The cassettes were sealed during transport. In the laboratory, the samples were conditioned and weighed using an XA 110 analytical balance (Radwag, Radom, Poland) with accuracy (d) within 0.01 mg. The dust concentration was calculated as the ratio of the weight of dust retained in the filters to the volume of filtered air. Microclimatic parameters, i.e. temperature, humidity, and air movement, were measured during the study using a thermohygrometer (Termo-higrometr AB-3321, Abatronic Sp. zo.o., Radom, Poland) and an anemometer (Hot Wire Anemometer AM-4204, Lutron, Taipei, Taiwan).

Biological aerosol in the stables and riding halls was measured using a MicroBio MB1 sampler with a 220-hole head. There were 6 measurement points at each site, and the measurements were repeated at each point. Sampling Volume per Sample was set at 100 L of air (De Ville Biotechnology, Raszyn, Poland). Following incubation of plates, the results were expressed as colony forming units (CFU) per m^3^ of air. For the quantitative assessment of bacterial aerosol, TSA (tryptone-soybean-agar; BTL Polska Sp. z o.o.) was used, MEA (Malt Extract Agar; BTL Polska Sp. z o.o.) was used for fungal aerosol, and MacConkey medium for coliform bacteria was used. Moulds were identified using microcultures on the basis of keys [17–21]. The percentage shares of the taxa identified were calculated, assuming that the total number of fungi was 100%. Analysis of bacterial aerosol was based on quantitative determination of CFU/m^3^.

### Statistical analysis

Statistical analysis of all results was performed using Statistica software ver. 13.3. Mean values were determined for each site. The results were given as means (M), standard deviation (SD) and standard error of the mean (SEM). Analysis of variance of the data was performed, checking the normality of the distribution, and the level of statistical significance was adopted for *p* < 0.05. For data that were not normally distributed, the criteria for ANOVA were not met, so Kruskal–Wallis ANOVA was used. Statistically significant values were designated with different letters (a, b…). MS Excel Solver as a tool that can be used to find the optimum value, which can be the maximum or minimum, was used to model dust concentrations in the stables in relation to the acceptable limits in Poland has been determined to be 3 mg/m^3^. This value satisfies animal welfare requirements, including microclimate conditions, and is given by [1].

## Results

### Concentrations of gaseous pollutants in the air

The results obtained for the concentrations of gaseous pollutants in the air at sites A, B, and C are presented in Table 1. The highest concentration for hydrogen sulphide was obtained in stable C (33.87 µg/m^3^). The lowest values for this pollutant were recorded in stable A (17.26 µg/m^3^; Table 1).

Among the inorganic compounds identified in the air at the sites, the highest concentration was obtained for ammonia in riding hall C (0.43 mg/m^3^; Table 1). Among the inorganic substances identified in the air of the stables, the highest level was obtained for ammonia in stable C (22.39 mg/m^3^), as in the case of the riding halls (Table 1).

**Table 1:** Concentrations of inorganic pollutants in the air of the riding halls and stables at each site (mg/m^3^; *N* = 12)

Pollutant		Ammonia (mg/m^3^)	Hydrogen sulfide (mg/m^3^)	Nitrates (mg/m^3^)	Nitrites (mg/m^3^)	Chlorides (mg/m^3^)	Sulphates (mg/m^3^)	
Site A	Stable	3.63 ± 2.45a	17.26 ± 1.33a	0.47 ± 0.05a	0.13 ± 0.05a	0.43 ± 0.33	0.4 ± 0.22c	
	Riding hall	0.16 ± 0.04a	26.08 ± 1.1a	0.13 ± 0.04	0.13 ± 0.04	0.16 ± 0.04	0.16 ± 0.04	
Site B	Stable	16.37 ± 1.99b	32.24 ± 2.96b	0.53 ± 0.05ab	0.17 ± 0.05ab	0.5 ± 0.29	0.63 ± 0.25	
	Riding hall	0.37 ± 0.05b	60.07 ± 2.36b	0.17 ± 0.05	0.13 ± 0.05	0.17 ± 0.05	0.14 ± 0.05	
Site C	Stable	22.39 ± 3.26c	33.87 ± 1.3bc	0.57 ± 0.05b	0.23 ± 0.05b	0.17 ± 0.05	0.57 ± 0.05a	
	Riding hall	0.43 ± 0.12bc	31.78 ± 1.76c	0.20 ± 0.08	0.13 ± 0.05	0.13 ± 0.05	0.13 ± 0.05	
SEM	Stables	2.46	2.32	0.02	0.02	0.08	0.06	
	Riding halls	0.04	4.51	0.02	0.01	0.01	0.01	
*p*-value	Stables	*p* < 0.001	*p* < 0.001	*p* < 0.05	*p* < 0.05	ns	ns	
	Riding halls	*p* < 0.01	*p* < 0.001	ns	ns	ns	ns	

*M ± SD, SEM – standard error of the mean, a, b, c – values with different letters are significantly different at p < 0.05, ns – not significant; N- number of samples; test ANOVA was used for all trials*

### Dust concentration in the air

**Fig. 1:**
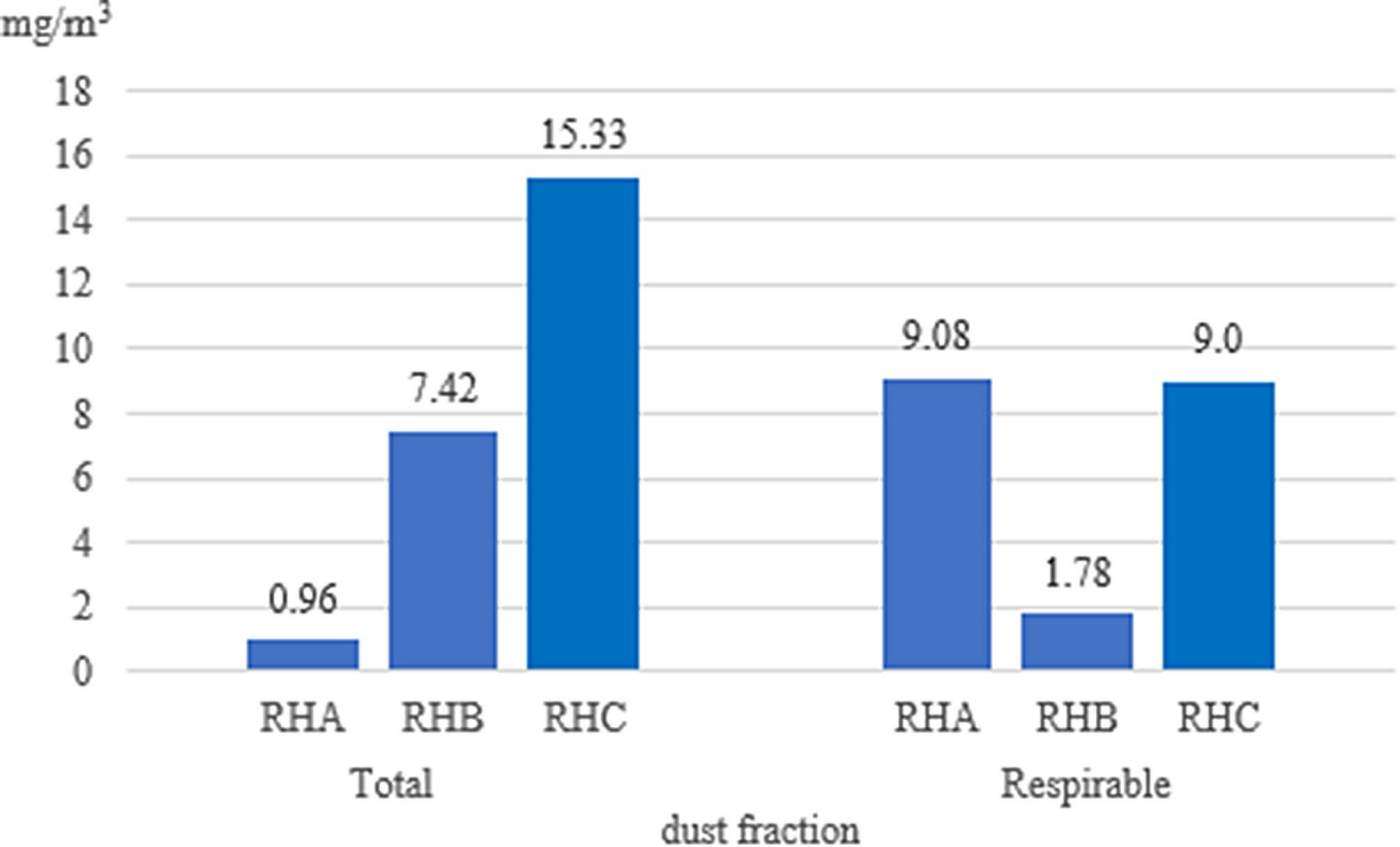
Average dust concentration in riding halls (mg/m^3^) (RHA, RHB, RHC - individual riding halls)

The measurements of dust concentrations took into account total and respirable dust. In the riding halls, the highest total dust concentration was noted in riding hall C (15.33 mg/m^3^), and the lowest in riding hall A (0.96 mg/m^3^). In the case of respirable dust, the highest concentration was noted in riding halls A and C (9.08 mg/m^3^ and 9 mg/m^3^, respectively), and the lowest in riding hall B (1.78 mg/m^3^; Fig. 1).

**Fig. 2:**
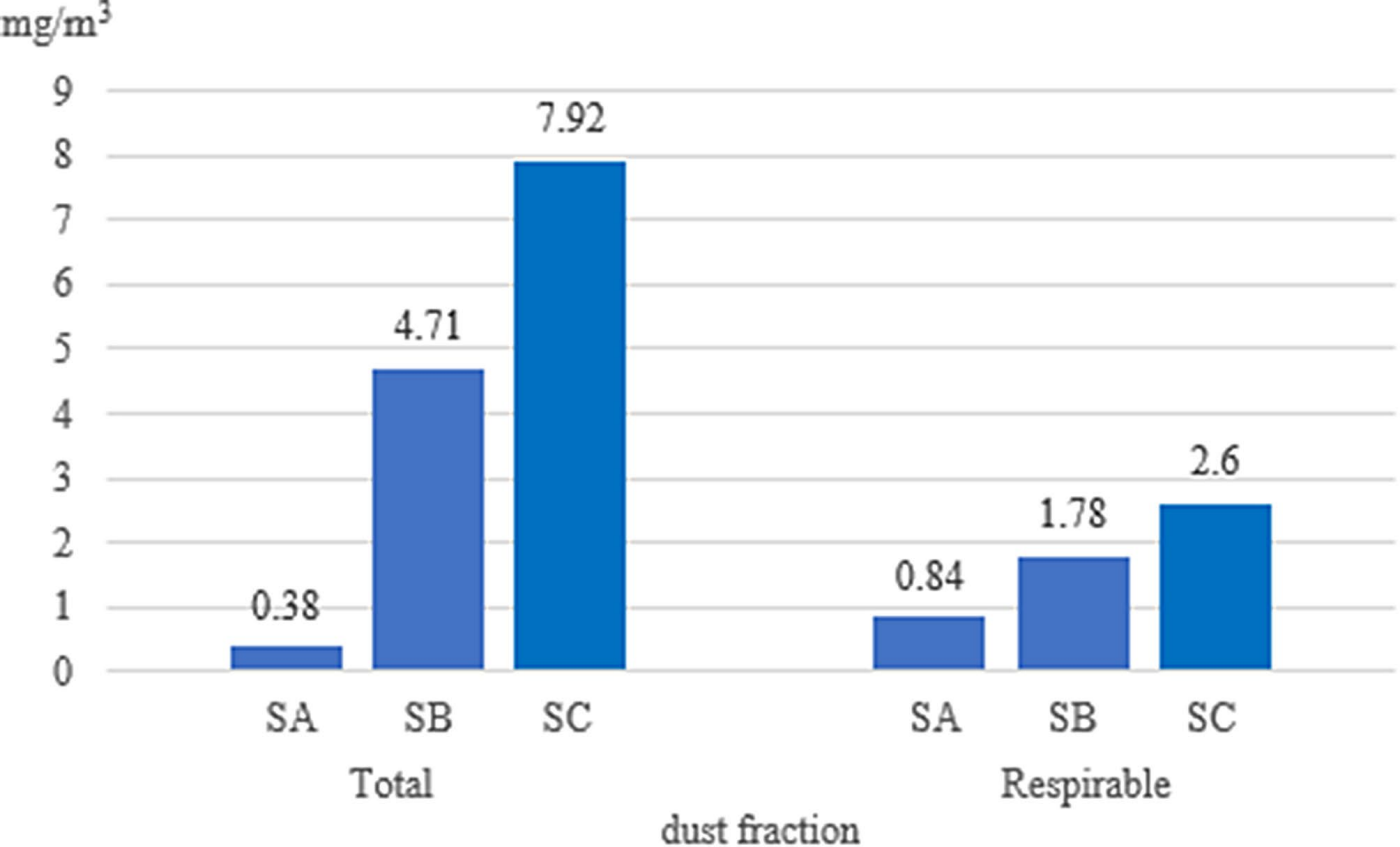
Average dust concentration in stables (mg/m^3^) (SA, SB, SC - individual stables)

In the stables, the highest total dust concentration was noted in stable C (7.92 mg/m^3^), and the lowest in stable A (0.38 mg/m^3^). In the case of respirable dust, the highest level was obtained in stable C (2.61 mg/m^3^), and the lowest in stable A (0.84 mg/m^3^; Fig. 2).

For comparison with acceptable limits for horses, modelling in MS Excel Solver was used to determine the length of time that was safe for horses to remain in the buildings (Tables 2 and 3). The dust concentrations allow horses to remain safely in riding hall A for 52 min, but only for a few minutes in riding halls B and C (Table 2).

**Table 2:** Dust concentrations in riding halls modelled in MS Excel Solver in relation to acceptable limits (3 mg/m^3^)

Riding hall	Total dust		Respirable dust	
	Average dust concentration	max. time spent in the site [min]	Average dust concentration	max. time spent in the site [min]
A	0.96	52	9.08	5
B	7.42	6	1.78	28
C	15.33	3	9	5

**Table 3:** Dust concentrations in stables modelled in MS Excel Solver in relation to acceptable limits (3 mg/m^3^)

Stable	Total dust		Respirable dust	
	Average dust concentration	max. time spent in the site [min]	Average dust concentration	max. time spent in the site [min]
A	0.38	131	0.84	59
B	4.71	10	1.78	28
C	7.92	6	2.61	19

For comparison with acceptable limits for horses, modelling in MS Excel Solver was used to determine the length of time that was safe for horses to remain in the stables (Table 3). Horses can remain safely in stable A for 131 min (about 2 h), but for only a few minutes in stables B and C (Table 3).

Comparison of the results reveals a significant problem arising from the high dust concentration in the stables in relation to the time the horses spend in them.

### Bioaerosol

The results obtained for the bioaerosol concentrations in the air of the stables are presented in Table 4. The highest number of bacteria was noted in stable B (1.05 × 10^5^ CFU/m^3^), and it was statistically significantly higher than in stable A (6.4 × 10^4^ CFU/m^3^). On the other hand, the number of fungi was higher in the air of stable C (6.2 × 10^3^ CFU/m^3^). The number of bacteria was highest in the air in riding hall B (5.8 × 10^4^ CFU/m^3^) and was statistically significant at *p* < 0.007 (Table 4).

**Table 4:** Average bioaerosol concentration in the stables and riding halls [CFU/m^3^]

Parametr	Site A		Site B		Site C		SEM		*p*-value	
	Stable	Riding hall	Stable	Riding hall	Stable	Riding hall	Stables	Riding halls	Stables	Riding halls
Total bacterial count	6.4 × 10^4^ab	2.2 × 10^3^a	1.05 × 10^5^a	5.8 × 10^3^b	4.97 × 10^4^b	2.2 × 10^4^c	9483.07	7283.03	*p* < 0.01	*p* < 0.001
Total fungal count	4.53 × 10^3^	2.0 × 10^2^	3.03 × 10^3^	1.7 × 10^3^	6.15 × 10^3^	1.3 × 10^3^	867.28	303.39	ns	ns
Coliforms	2.5 × 10^2^	No growth	1.8 × 10^2^	1.0 × 10^2^	1.0 × 10^2^	1.0 × 10^2^	30.46	22.47	ns	ns

*SEM – standard error of the mean, a, b, c – values with different letters are significantly different at p < 0.05, ns - not significant; test ANOVA was used for all trials except Total bacteria and fungal in stables and Coliforms for riding halls for which Kruskal–Wallis ANOVA was used*

Among the moulds identified, *Ulocladium* sp. accounted for the highest percentage. This percentage was highest in stable A (83.33%) and riding hall A (50.0%; Table 5).

**Table 5:** Percentages of mould taxa in air samples from the buildings (%)

Identified moulds	SA	SB	SC	RHA	RHB	RHC
*Aspergillus* spp.	-	-	-	33.33	38.10	40.00
*Fusarium* spp.	5.56	8.33	15.38	-	4.76	10.00
*Mucor* spp.	5.56	8.33	7.69	16.67	9.52	30.00
*Rhizopus* spp.	-	2.78	7.69	-	-	-
*Trichoderma* spp.	-	2.78	11.54	-	-	-
*Ulocladium* spp.	83.33	77.78	57.69	50.00	47.62	20.00
Unidentified	5.56	-	-	-	-	-

### Temperature and humidity

Microclimatic parameters (temperature, relative humidity, and air movement) were measured in the stables and riding halls during the study. The results are presented in Table 6. The air temperature in the stables ranged from 4.63 °C (B) to 8.07 °C (A), with relative humidity from 71.57% (C) to 74.30% (B) and air movement from 0.12 m/s^2^ (A) to 0.27 m/s^2^ (C). In the riding halls the air temperature was between 3.5 °C (C) and 8.77 °C (B), with relative humidity from 53.22% (B) to 73.53% (A) and air movement from 0.23 m/s^2^ (A) to 0.3 m/s^2^ (C) (Table 6).

**Table 6:** Microclimatic parameters in the stables and riding halls (*N* = 12)

Parameter	Stable	M	SEM	*p*-value
Temperature [°C]	SA	8.07a	0.754	*p* < 0.01
	SB	4.63b	0.118	
	SC	6.30ab	0.579	
	RHA	4.05a	0.28	*p* < 0.001
	RHB	8.77b	0.32	
	RHC	3.5ac	0.26	
Relative humidity [%]	SA	73.77	1.631	ns
	SB	74.30	4.551	
	SC	71.57	1.6	
	RHA	73.53a	1.71	*p* < 0.001
	RHB	53.22b	1.4	
	RHC	70.23ac	1.94	
Air movement [m/s]	SA	0.12a	0.008	*p* < 0.05
	SB	0.20ab	0.041	
	SC	0.27b	0.024	
	RHA	0.23	0.03	ns
	RHB	0.27	0.04	
	RHC	0.3	0.03	

*M – mean, SEM – standard error of the mean, a, b, c – values with different letters are significantly different at p < 0.05, ns - not significant, N- number of samples; test ANOVA was used for all trials except Relative humidity in stables and Air movement for riding halls for which Kruskal–Wallis ANOVA was used*

## Discussion

Air quality is a very important factor influencing the occurrence of respiratory disease in horses, hence the high level of interest in the effect of the floor material in the riding hall where training takes place and bedding in the boxes where the horses rest [22]. White et al. [23] report that there are numerous allergens in the horse’s environment which can lead to severe equine asthma (sEA). Asthma in horses limits their use. Air quality is determined by numerous factors, including the quality of the feed, the type of bedding, the floor material used in riding halls and how often it is changed, and maintenance of cleanliness in the boxes and paddocks [24, 25]. Relationships are observed between the presence of organic and respirable dust and chronic bronchitis in riding instructors, which suggests that horses can be an excellent model for assessment of the quality of this environment [26]. Particular attention should be paid to the riding hall, where during increased physical activity the animal takes in more air, and with it increased levels of harmful chemical and biological agents. This problem concerns artificial surfaces as well [17, 26, 27]. In stables during winter, the relative humidity should not exceed 80%, assuming that the temperature in the stable is less than 10^o^C [28]. In studies by Elfman et al. the average level of dust concentration was 0.6 mg/m^3^ [28]. Referring to the Swedish guidelines for horses (10 mg/m^3^), the obtained total dust values in our own research did not record any exceedances of these values, with the exception of riding school C [29].

According to the European Committee for Standardization (Comité Européen de Normalization), the smaller the particle size (< PM 0.1), the greater risk the particles pose to the body, as they diffuse to the lower respiratory system and penetrate the alveoli. Therefore, assessment of biological aerosol and dust concentrations in stables and riding halls must take into account the size of the particles in the air. Wheeler et al. [30], in a study conducted in equestrian arenas, showed that in a riding hall with a sand floor, 60% of the dust collected was respirable. In riding halls, A and C, the percentage of respirable dust during training of horses was higher than at site B. Gesche and Engel [17] demonstrated interactions between dust concentrations and the month of the year as well as the time spent by the animals in the riding hall. The authors point out that moistening the surface can reduce the dust concentration in the air.

The German Senate Standing Committee on the Investigation of Harmful Working Materials (Ständige Senatskommission zur Prüfung gesundheitsschädlicher Arbeitsstoffe) gives normative values for individual dust fractions. The acceptable concentration of the respirable fraction of granular dust was determined to be 0.3 mg/m^3^, while the limit for the total dust concentration is 4 mg/m^3^. In the present study, these recommended levels were exceeded in stables B and C. Using the MS Excel Solver model in relation to the acceptable levels, safe time periods for horses in air with sustained long-term dust concentrations were obtained. The presence of suspended particulates (PM 10 and PM 2.5) in the horses’ breathing zone is conducive to the accumulation of mucus in the trachea and an increase in inflammatory cells. In addition to dust, *Penicillium* spp., *Cladosporium* spp. and *Aspergillus* spp. are often suspended in the air [16, 17, 31]. For this reason, it is important to pay attention to the air exchange in the stable and, at the planning stage, to the orientation of the stable. In the present study, *Ulocladium* spp. and *Aspergillus* spp. had the highest percentage shares of the moulds. Moulds of the genus *Ulocladium* spp. are found in feed, air, soil, and even building materials, carrying out biodeterioration [32]. The presence of this biological aerosol depends on the quality of feed, the sanitary condition of the facilities, and microclimatic conditions. Due to the use of horses for sport and recreational purposes, there is a need to monitor the air quality, especially during training sessions in the riding hall, in order to reduce the horses’ exposure to dust particles on which bioaerosol is suspended. Keeping in mind that workers are also exposed to these pollutants [33]. Optimal microclimatic conditions positively influence the welfare of horses. These levels were maintained in the present study [34].

It should be borne in mind, however, that ammonia is generated from the degradation of nitrogen compounds in manure and from conversion of urea by urease and microorganisms present in bedding [20]. According to Elfman et al. [28], Ivester et al. [11], and Saastamoinen et al. [35], the ammonia concentration in the building is influenced by the bedding in the stable and the floor material in the riding hall. Inhalation of ammonia by horses, especially those used for sport and recreation, can lead to conjunctivitis and also increases susceptibility to respiratory disease. At low concentrations, ammonia mainly affects the upper respiratory tract, while increased concentrations and prolonged exposure lead to metabolic disorders of the liver and secretion in the form of urea. Intensive use of horses, especially sport horses, can furthermore lead to oxidative stress and have a negative impact on welfare [36].

Biological (fungi) and chemical agents (PM 10, PM 2.5, NO, NO_2_, and SO_2_) in the air play a major role in diseases of the lower and upper respiratory tract. Training causes the horse to take in more air, and physical exertion results in bronchodilation and an increase in the respiratory rate and tidal volume. NO plays an important role in regulating airway function by signalling that the airways are relaxed. It also regulates the animal’s immune response and controls the release of various inflammatory substances involved in inflammatory responses. Their concentration in the air may be a biomarker of the respiratory [37–39]. Araneda [40] observed that air pollution affects the performance of horses used for sport and draws attention to a wide range of effects of inhaled air components on the body. According to Whittaker et al. [41] in stables and riding halls with an efficient ventilation system the main factor determining exposure to air pollutants and respirable dust is the floor material and feed [42]. Mönki et al. [22] demonstrated that the floor material and suspended compounds in dust are significantly linked to the occurrence of equine asthma in horses. Similar results were obtained by Fleming et al. [43] in a study of respirable dust from various floor materials. PM 10 and PM 2.5 levels were highest for litter consisting of hemp and flaxseeds and lowest for straw pellets, wood shavings, paper cuttings, and straw. In the present study, the highest total dust concentration was obtained in the riding halls using sand, sawdust and gravel (RHB and RHC), as confirmed by Wheeler et al. [30]. The authors report that increasing the moisture in the floor material in the riding hall improves air quality.

Mönki et al. [44] found that peat bedding causes fewer cases of respiratory inflammation in horses than wood shavings. An increase in the concentration of pollutants in the air increases inhalation exposure in horses. According to Whittaker et al. [41] the respirable dust fraction in the stable can account for 30% to even 60% of total dust, and the indoor dust concentration increases in winter [11] which is in agreement with the present study.

In the literature on maintenance conditions for horses used for recreation, there are few studies on the problem of the accumulation of inhalation hazards in stables, not only microbiological contaminants, but also chemical contaminants and dust. The synergistic effect of these factors, however, is of great importance in animals such as horses, which have a sensitive respiratory tract. Among the many chemical compounds identified, the respiratory system of horses is most threatened by ammonia, which can synergistically magnify the toxic effect of the high dust concentrations in the air. The other gaseous pollutants identified (trace gases) may enhance this harmful effect. Animal welfare may be additionally harmed by high concentrations of microscopic fungal spores, which are one of the main groups of inhalant allergens. Given the existing threat to the respiratory system of horses, there is a need for monitoring and for measures to improve air quality through the choice of bedding material and hygiene in indoor facilities.

## Conclusions

Among the chemical air pollutants identified, ammonia remains the gas of greatest concern. Its concentration is variable and depends on both management and conditions of horse maintenance. The problem of harmful gaseous pollutants may be compounded by an increase in total dust concentrations, exceedances of which have been recorded in both the stable and the C riding arena. According to the study, the age of the facility is an important factor affecting the condition and degree of dust and chemical pollution. Concentrations of bacterial and fungal aerosol obtained the highest values precisely in the oldest facility. The results indicate that it is necessary to modernize the facilities where horses are housed, appropriate selection of the substrate in the dressage halls to avoid respiratory health problems during the use of animals. In addition, when planning the recreational use of horses, it is advisable to control the microclimatic conditions in the premises to maintain the welfare of the horses.

## Data Availability

Most of the data generated or analysed during this study are included in this published article. The rest of data is available from the corresponding author on reasonable request.
